# Surgical safety and personal costs in morbidly obese, multimorbid patients diagnosed with early-stage endometrial cancer having a hysterectomy

**DOI:** 10.1186/s40661-016-0023-8

**Published:** 2016-02-09

**Authors:** Andreas Obermair, Donal J. Brennan, Eva Baxter, Jane E. Armes, Val Gebski, Monika Janda

**Affiliations:** Queensland Centre for Gynaecological Cancer, The University of Queensland, Brisbane, QLD Australia; Greenslopes Private Hospital, Brisbane, QLD Australia; Rotunda Hospital, Dublin, Ireland; QIMR Berghofer Medical Research Institute, Brisbane, QLD Australia; Anatomical Pathology Mater Health Services, Mater Adult Hospital, and Mater Research Institute-University of Queensland, Brisbane, QLD Australia; University of Sydney NHMRC Clinical Trials Centre, Sydney, NSW Australia; School of Public Health, Institute for Health and Biomedical Innovation, Queensland University of Technology, Brisbane, QLD Australia; Queensland Centre for Gynaecological Cancer, c/o Royal Brisbane and Women’s Hospital, Butterfield Street, Herston, Brisbane, QLD 4029 Australia

**Keywords:** Endometrial cancer, Endometrial hyperplasia with atypia, Obesity, Surgery, Adverse event

## Abstract

**Background:**

Many women who develop endometrial cancer (EC) or endometrial hyperplasia with atypia are obese and therefore at high risk of surgical complications. Recently clinical trials have been initiated offering non-surgical treatment to these women, but not all may agree to participate in such trials. This paper aims to describe the patient characteristics, and surgical outcomes of women with suspected early stage endometrial cancer and body mass index (BMI) of 30 or greater, who declined enrolment in the feMMe trial, which offers non-surgical hormonal treatment, hormonal plus metformin or hormonal plus weight loss as primary treatment.

**Methods:**

Consecutive case series from a tertiary gynaecological oncology unit. Over the course of the first 2 years of the feMMe trial, 27 patients met the initial eligibility screening, but declined enrolment in the feMMe trial and opted for upfront surgery. The main surgical outcome measures were type of surgical approach, need for conversion from laparoscopic to open approach, length of stay in hospital and adverse events.

**Results:**

Patients’ median age was 63 years (range 40 to 86); median BMI was 37.3 kg/m2 (range 30.7 to 54.7); median medical co-morbidities were six (range 3–10). Of the 26/27 surgeries planned to be undertaken laparoscopically, 2/26 patients had to be converted (7 %). Overall, the average hospital stay was 4.5 days, and 11/27 (41 %) of the patients developed one or more adverse events grade 2+ rated according to the Common Toxicity Criteria Version 3.

**Conclusions:**

Adverse surgical outcomes are common in multi-morbid, obese or morbidly obese patients diagnosed with early stage EC or endometrial hyperplasia with atypia and who have a hysterectomy.

## Background

Obesity is a massive health issue in many countries around the world and is also the major risk factor for Endometrial Cancer (EC) [1–4]. It has previously been reported that obesity causes at least 39 % of cases of EC [5]. Obesity is also associated with increased risk of medical co-morbidities (e.g., diabetes, cardiovascular) [6–8]; the need for intense preoperative assessments; perioperative complications [9]; conversion from laparoscopic to open surgery [10]; intensive postoperative care [11, 12]; treatment costs [11, 13]; and reduced recurrence-free survival [9, 14, 15].

While surgical treatment of EC is generally effective, it does not address the specific needs of the steadily growing group of morbidly obese and multi-morbid patients as well as young obese patients still desiring fertility [16, 17]. For these growing groups of patients treatment often comes at a high personal cost (long hospital stay, protracted recovery from surgery, high incidence of postoperative complications) and subsequent high healthcare cost. We previously estimated hospital costs of $12,872 vs $25,652 for patients without or with a surgical complication, respectively [11].

The challenges of pre- and postoperative care for multi-morbid and morbidly obese EC patients impact on a variety of resources. Thus, as highlighted by the Royal College of Obstetricians and Gynaecologists, treatment for EC needs to be reassessed in this complex and increasingly common situation [18]. The search for treatment alternatives that are safe, effective and less harmful than surgery is warranted.

Recently, the Gynecologic Cancer InterGroup identified conservative treatment for fertility sparing purposes and to treat morbidly obese women as a most pressing research priority at their EC Clinical Trials Planning Meeting in the Netherlands [19].

To address this need the feMMe trial was initiated in 2013 [20]. It is an open-label, randomised clinical trial exploring conservative, non-surgical treatment options to achieve a pathological complete response in patients diagnosed with early-stage EC (ANZGOG #1301, NCT01686126).

The aim of the present study was to describe the safety and clinical outcomes of consecutive patients who would have fulfilled the eligibility criteria for the feMMe trial and who were offered participation in the feMMe trial, but declined enrolment and opted for hysterectomy at two institutions instead.

## Methods

Approval for this study was received from the Royal Brisbane & Women’s Hospital Human Research Ethics Committee (HREC/15/QRBW/113). All patients reported here have been identified through gynaecological oncology services at Royal Brisbane and Women’s Hospital and Greenslopes Private Hospital. These patients would have been considered potentially eligible to be enrolled in the feMMe trial.

The feMMe trial is an open label, randomised phase II trial with three treatment arms and is recruiting patients at present [20]. The three arms consist of Intrauterine Progestin (IUP) placed into the uterine cavity (45 patients); IUP plus Metformin 1000 mg daily (75 patients); or IUP plus weight loss through Weight Watchers (45 patients). Weight Watchers is a standardised, evidence-based and formally tested weight loss intervention including diet, physical activity, social networking and support via a network of lifestyle centres, one-on-one support and an online program [21, 22]. It has been shown to be the most cost-effective among a range of currently available weight loss programs [22].

In this phase II trial randomisation aims to eliminate selection bias rather than allow a formal comparison of groups. Trial methodology, in-/exclusion criteria, randomisation /stratification and study assessments were published recently [20]. Human Research and Ethics Committee and site-specific approvals are underway in various Australian States but only sites in the state of Queensland are fully approved and enrolling at present. All patients are followed for 6 months and a central pathology review will be conducted once all patients are enrolled.

Eligibility criteria: Only patients with histologically confirmed innocuous EC or endometrial hyperplasia with atypia, Body Mass Index (BMI) >30 kg/m2 who wish to retain fertility or who suffer from medical impairments and are considered suboptimal candidates for hysterectomy are eligible [20].

Patients are excluded from enrolment if they had a histological type other than endometrioid adenocarcinoma of the endometrium, clinically advanced disease, involvement of the uterine cervix or enlarged retroperitoneal lymph nodes.

To be eligible for this study, patients had to have a CT scan of the abdomen and pelvis as well as imaging (CT or X-Ray to the chest) suggesting the absence of extrauterine disease. Patients are also only considered eligible if their baseline serum CA-125 reading was 30 U/ml or less.

Patients who agree to proceed with enrolment into the feMMe trial receive a pelvic MRI to ensure that the depth of invasion is not greater than 50 % of myometrium and to re-confirm the absence of extrauterine dissemination. Patients who decline participation in feMMe (including the patients reported herein) however do not receive an MRI as it is not part of the standard imaging workup in our institutions.

In addition to the established criteria for low-risk disease (CT and MRI scan showing the absence of extrauterine disease, FIGO grade = 1) we offer enrolment only to patients with serum CA125 of 30 U/ml or less [23]. Considering the strict criteria above, we expect that the risk of enrolling patients with advanced or aggressive disease is minimal.

All patients reported here did not qualify for feMMe because they preferred hysterectomy and as a consequence declined enrolment into the feMMe trial. Hence, following standard protocols, a pelvic MRI was not offered (as has been explained above).

In all 27 patients, pre-existing medical co-morbidities were recorded as well as any intra- or post-operative Adverse Events (AEs) up to 30 days post-operatively. We coded AEs according to the post-operative Common Toxicity Criteria (CTC) Version 3 and report any AEs grade 2+ (moderate to severe AEs). Analyses were restricted to women who completed 30 days of follow-up after surgery.

## Results

The clinical outcomes of 27 patients who fulfilled the eligibility criteria but declined participation in the feMMe trial and have chosen primary surgical treatment instead are reported here.

Patients’ median age was 63 years (range 40 to 86 years) and the median BMI was 37.3 kg/m2 (range 30.7 to 54.7 kg/m2), median ASA was 3 (range 2–3). At baseline a total of 167 medical co-morbidities were recorded among the 27 patients including hypertension, hyperlipidemia, hypercholesterolemia, diabetes, obstructive sleep apnoea, fatty liver and many other lifestyle-related ailments.

Twenty-six patients had a total laparoscopic procedure of which two patients (7 %) had to be converted to a laparotomy. One patient required an abdominal hysterectomy through a midline incision (Table 1).

**Table 1:** Patient characteristics

Patient #	Age (years)	D&C histology	BMI	Surgical approach	Node dissection	LOS	Stage	FIGO grade	Depth of invasion (%)	Intra-, Postoperative complications
1	40	EHA	31.0	TLH	0	2	1a	1	15	
2	56	G1 EAC	49.2	TLH	0	1	1a	1	0	Post-operative bleeding
3	62	G1 EAC	35.1	TLH	0	2	1a	2	11	
4	69	G1 EAC	54.7	TLH	0	2	1a	2	38	Vault haematoma, abdominal cramping, urinary frequency
5	75	G1 EAC	39.6	TLH	0	2	1a	1	0	
6	65	G1 EAC	40.9	TLH	0	2	1b	2	60	
7	56	G1 EAC	42.7	TLH	0	5	1a	1	0	
8	74	G1 EAC	46.8	TLH	0	1	1b	2	52	
9	73	G1 EAC	44.8	TLH	0	8	2	2	18	Post-operative bleeding, anaemia, retroperitoneal haematoma, rise in Troponin
10	68	EHA	35.4	TLH	1	2	1b	2	60	
11	75	G1 EAC	38.9	TLH converted to TAH	0	14	1b	2	57	Unplanned gastrostomy, unplanned stay in ICU
12	69	EHA	45.5	TLH	0	2	1a		0	
13	67	G1 EAC	32.5	TLH	0	3	1a	2	8	Vault haematoma, hypokalaemia, sinus tachycardia
14	57	G1 EAC	30.7	TLH	0	2	1a	1	50	Vault haematoma, pain
15	73	G1 EAC	34.3	TLH	0	5	1a	1	10	
16	69	EHA	29.4	TLH	0	2	1a	1	0	Vault haematoma, hypotension, hypokalaemia
17	68	G1 EAC	43.9	TLH	0	2	1a	2	45	
18	78	G1 EAC	32.0	TLH	0	2	1a	1	45	
19	63	EHA	33.5	TLH	0	2	0		0	Hypertension
20	66	EHA	40.6	TLH	0	2	1a	1	1	Atrial fibrillation
21	60	EHA	37.1	TLH	0	7	1a	1	37	
22	70	EHA	43.1	TLH	0	5	1b	2	75	Chest infection, wound dehiscence, vault haematoma, pain
23	72	G1 EAC	42.7	TLH	1	3	3b	1	100	
24	50	EHA	35.6	TLH	0	9	1a	1	39	Fluid overload, pulmonary oedema
25	55	G1 EAC	33.3	TAH	0	4	2	1	7	
26	73	G1 EAC	47.0	TLH	0	2	1a1	1	0	
27	86	G1 EAC	43.6	TLH converted to TAH	1	28	1a	1	31	Unplanned enterotomy, wound infection, Atrial fibrillation, renal failure

*Abbreviations*: *BMI* body mass index, *EAC* endometriod adencocarcinoma, *EHA* endometrial hyperplasia with atypia, *TLH* total laparoscopic hysterectomy, *TAH* total abdominal hysterectomy, *LOS* length of stay, *FIGO* the international federation of gynecology and obstetrics

The reasons for conversion to open surgery in two patients included an inadvertent gastrotomy through a trocar at primary port entry, which required primary surgical closure. The second patient sustained an enterotomy to the small bowel during adhesiolysis. The adhesions could not be dissected from the anterior abdominal wall laparoscopically. The enterotomy was recognised at surgery and the operation was completed through open surgery. The median percentage of invasion into the endometrium was 31 % (range 0–100 %).

The average postoperative hospital stay was 4.5 days (median 2 days), ranging from 1 to 28 days. The patient with a 28-day hospital stay was a patient with a body mass index of 43.6 kg/m2 who required conversion from laparoscopic to open surgery. She developed a wound infection (limited to the subcutaneous adipose tissue), atrial fibrillation resulting in a stay at the Cardiac Care Unit followed by acute renal failure. The patient was discharged into rehab on day 28 post surgery.

Within 30 days from surgery, 12 patients developed at total of 30 AEs. One of 27 patients developed an AE CTC grade 1 and 11/12 patients developed one or more AEs CTC grade 2+ (41 %). All but 5 AEs were surgery related (Table 1).

Nine patients were enrolled to treat endometrial hyperplasia with atypia based on a pre-hysterectomy endometrial biopsy or curette and 18 of 27 patients had surgery for histologically proven endometrioid endometrial adenocarcinoma on endometrial biopsy or curette.

Of those nine patients who were treated for endometrial hyperplasia with atypia, seven patients were found to have endometrial adenocarcinoma in the final histopathology specimen of the uterus.

In patients with the final histopathological outcomes confirming endometrial adenocarcinoma, all patients were diagnosed with endometrioid cell type. In those patients FIGO grade was grade 1 in 14/25 patients and grade 2 in 10/25 patients. In one patient there was no residual disease at hysterectomy. The depth of invasion was limited to inner half in all but five patients.

Two patients had extension of disease into the endocervix (stage 2) and one patient had full thickness myomterial invasion of a grade 1 adenocarcinoma and focal involvement of a fallopian tube (FIGO stage 3b).

## Discussion

### Main findings

Adverse surgical outcomes are common in multi-morbid and morbidly obese patients diagnosed with early stage EC who have a hysterectomy. Obesity is an independent risk factor for AEs, regardless of the surgical approach [12]. Obese women will have a higher risk of conversion to open surgery [10] and their risk of surgical AEs is higher [24].

For comparison we quote data from the prospective randomised and multi-institutional LACE trial below [12, 25]. The LACE trial compared open with laparoscopic surgery for early stage EC or endometrial hyperplasia with atypia. It was an international trial but the vast majority of patients were treated in Australian institutions.

In the case series reported here, all but one operations were planned to be performed laparoscopically; two of the 27 patients required a conversion from laparoscopy to laparotomy (7 %) and one patient required a primary laparotomy, implying that 10 % of patients required a laparotomy to accomplish the surgical task of a hysterectomy. By contrast, the conversion rate from laparoscopic to open in the prospective randomised and multi-institutional LACE trial was only 3.8 %, most likely due to omitting the requirements for a comprehensive pelvic and aortic retroperitoneal node dissection in these patients and a smaller proportion of patients with a BMI of 30 or greater.

By contrast, pelvic and aortic lymph node dissection was mandatory in the LAP-2 trial corresponding to a 25.8 % conversion rate. Patients with a high BMI had an up to 60 % risk of conversion to open surgery [24].

In the context of morbidly obese and multi-morbid patients we typically aim to minimise the risk of conversion, which may attract further intraoperative and postoperative morbidity. In those instances patients’ adjuvant treatment may be guided by their general medical health and histopathological features available from the primary tumour. At present only low-level evidence is available on the feasibility and safety of robotic surgery in morbidly obese patients requiring a retroperitoneal node dissection. Deaths due to complications of robotic surgery have also been reported [26].

In this sample, the mean length of hospital stay (LOS) was 4.5 days. Length of stay was largely associated with the development of postoperative complications. However, in some patients an uneventful postoperative recovery still required a longer than expected LOS due to slow recovery. In the LACE trial, reflecting the Australian health care situation the LOS was 2.4 days for patients assigned to have a laparoscopic hysterectomy and 5 days for patients who were randomised to have a laparotomy.

In this series of patients the per-patient incidence of surgical AE’s CTC grade 2+ was high at 41 %. 

### Strength and limitations

Innovatively this case series details the outcomes of patients who were offered enrolment in a non-surgical clinical trial, but preferred surgery. These results again highlight the increased risk of obese patients to develop complications as previously shown in other international series. This group of morbidly obese and multi-morbid patients carries a high risk of conversion to laparotomy, a longer hospital stay and a three to four times higher risk of surgical AEs compared to previous series with a wider range of BMI. LOS and AEs are significant contributors to health care costs and funders of health care services must therefore expect high costs among obese patients treated surgically for EC [13]. Limitations of this study include the non-random assignment to surgery, which was based on patients’ preference, as well as the relatively small number of patients available for data collection.

## Conclusions

While hysterectomy for EC offers excellent survival outcomes, it also comes at a price: slow recovery from surgery, surgical AEs, loss of fertility, financial and societal treatment costs [27, 28].

Importantly these results indicate that current risk estimations do not take populations at high surgical risk (e.g. obese and multi-morbid patients) into sufficiently account. Thus, the efficacy of treatment alternatives need to be assessed in the complex and increasingly common situation of obesity and EC or endometrial hyperplasia with atypia [18]. We envisage that for obese and multimorbid patients less invasive treatments will achieve equivalent survival outcomes at a lower personal and financial cost to patients and society [13].

Historically intracavitary brachytherapy has been used to treat patients at advanced age and severe medical illnesses and with the advent of IMRT [29], radiotherapy may be well positioned to be evaluated in clinical trials as an alternative to major surgery.

By contrast, others units currently investigate the effectiveness of a levonorgestrel containing intrauterine device for the treatment of endometrial cancer. The feMMe trial (ANZGOG #1301, NCT01686126) is an international, phase II, 3-arm randomised clinical trial exploring conservative, non-surgical treatment options to achieve a pathological complete response in early-stage endometrial cancer patients who are suboptimal candidates for hysterectomy [20].

In addition to the eligibility criteria for this case series, patients have to have an MRI of the pelvis to determine the depth of myometrial invasion. In the context of the results reported here this is well warranted, and the pelvic MRI will also be critical to exclude involvement of the cervix and/or adnexae.

**Fig. 1:**
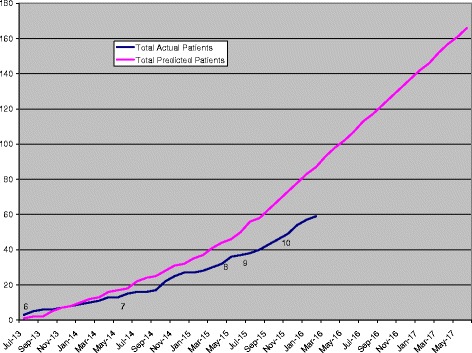
Recruitment chart for the FeMMe trial

The trial is recruiting at present (Fig. 1 shows the recruitment chart) and also includes a molecular component investigating the mechanisms of change associated with response or non-response to feMMe intervention (tumour polymorphisms; molecular phenotype of tumours; circulating cytokines, such as adipokines, hormones and growth factors). Phase II results are expected for 2017, and early discussion about the optimal Phase III trial design to follow have been initiated in 2015.

### Ethics approval

Royal Brisbane & Women’s Hospital Human Research Ethics Committee (HREC/15/QRBW/113).
